# Impact of a health system’s population health implementation of Medicare’s “Kidney Care First” program: a retrospective cohort study

**DOI:** 10.1186/s12913-026-15323-2

**Published:** 2026-08-17

**Authors:** William Turner, Manying Cui, Carlos Irwin Oronce, Chad Wes Villaflores, Samuel Skootsky, Sarah Meshkat, Richard Kemper Leuchter, Catherine Sarkisian, John N. Mafi

**Affiliations:** 1 Division of General Internal Medicine and Health Service Research, Department of Medicine, David Geffen School of Medicine, University of California, Los Angeles, 1100 Glendon Ave, STE 710, Los Angeles, CA 90024 USA; 2 UCLA Health Faculty Practice Group, Los Angeles, CA USA; 3 Geriatrics Research Education and Clinical Center (GRECC), VA Greater Los Angeles Healthcare System, Los Angeles, California USA; 4 RAND Corporation, Santa Monica, California USA; 5 Division of Hospital Medicine, VA Greater Los Angeles Healthcare System, Los Angeles, California USA

**Keywords:** Chronic kidney disease, Value based care, Kidney Care First, Population health, Care coordination, Acute care utilization, Medicare

## Abstract

**Background:**

Chronic kidney disease (CKD) affects more than one in seven US adults and accounts for a disproportionate share of Medicare expenditures. Medicare’s Kidney Care First (KCF) model represented a shift toward value-based care for patients with advanced CKD, yet evidence regarding its real-world effectiveness remains limited. We evaluated the impact of a population health intervention – the Kidney Care Medical Home (KCMH) program – on health care utilization outcomes in response to KCF.

**Methods:**

We conducted a retrospective cohort study of 926 KCF-enrolled and 551 control beneficiaries from 2021 to 2023. The intervention included complex care management, depression screening, kidney education, and emphasized referrals to transplant specialists and shared decision making on dialysis. We employed the Callaway and Sant’Anna difference-in-differences estimator to measure quarterly average treatment effects on the treated (ATET) for primary (all cause emergency department visits and hospitalizations) and secondary outcomes (home dialysis utilization, transplant nephrology referrals, and behavioral health referrals), adjusting for age, sex, social vulnerability index, dual eligibility, and comorbidities.

**Results:**

KCF enrollment was not associated with statistically significant reductions in all cause emergency department visits (quarterly ATET ranging from − 3.09 to + 2.80 per 100 patients) or hospitalizations (-3.00 to + 6.60 per 100 patients). However, home dialysis increased substantially by Q4 2023 (+ 4.96 per 100 patients), transplant nephrology referrals increased in Q3 2022 (+ 1.5 per 100 patients) and Q4 2022 (+ 1.7 per 100 patients), and behavioral health referrals increased by Q2 2022 (+ 3.9 per 100 patients).

**Conclusions:**

A comprehensive KCF-aligned care coordination program was not associated with short-term reductions in acute care utilization but was associated with meaningful improvements in home dialysis uptake, transplant referrals, and behavioral health engagement. Future payment models should consider aligned financial incentives, extended evaluation periods, and primary care coordination to realize the full potential of value-based kidney care in predominantly fee-for-service environments.

**Supplementary Information:**

The online version contains supplementary material available at https://doi.org/10.1186/s12913-026-15323-2.

## Background

More than 1 in 7 US adults have chronic kidney disease (CKD); and while they comprise 15% of the Medicare population [1], they account for 27% of total Medicare fee-for-service expenditures [2]. This disproportionate spending grew by 38% to $86.5 billion between 2012 and 2022 [2]. Patients with end stage renal disease (ESRD) account for an even more outsized cost: despite only comprising 0.24% of beneficiaries [2], their care accounts for 5.7% of total Medicare fee-for-service (FFS) expenditures ($28.0 billion) [2]. Much of this can be attributed to care delivery challenges: fragmented care between primary care and nephrology [3–9], low home dialysis initiation despite evidence linking it to superior outcomes [10], underscreening for depression [11], and late transplant referrals [2, 12].

Although observational studies have suggested better patient health outcomes, higher quality of life, and lower total expenditures associated with home dialysis (home-hemodialysis and peritoneal dialysis) [10, 13] compared with in-center hemodialysis, only 14.5% of new renal replacement therapy initiations include home dialysis [2]. Traditional FFS payment structures may contribute to high utilization of in-center hemodialysis [14–16]. 

Numerous policy initiatives and alternative payment models have attempted to address these disproportionate costs. The Comprehensive ESRD Care Model demonstrated modest but statistically significant improvements in health outcomes and total expenditures, but reflected a global payment program rather than focusing solely on CKD [17–19], whereas the ESRD Treatment Choices model directly incentivized home dialysis and demonstrated mixed results without addressing other components of CKD and ESRD care [20, 21]. The KCF Option reflected one of the most comprehensive programs financially incentivizing improvements in care quality and outcomes for Medicare FFS beneficiaries with advanced CKD (stages 4 and 5 and ESRD) [22, 23]. The KCF Option included a suite of capitated payments, pay-for-performance adjustments, and transplant incentives designed to optimize outcomes for patients and curb unnecessary utilization by incentivizing complex care management programs, optimal dialysis starts, and kidney transplants to reduce long term total cost of care [24]. However, the KCF Option was terminated early by CMS, lasting only 4 years after CMS reported approximately $304 million in net Medicare losses in performance year 2023; the accompanying model update also eliminated the kidney transplant bonus and cut by 50% the capitation payments across the remaining model options [23, 25]. Notably, CMS reported that the model improved quality through optimal ESRD starts, home dialysis rates, and preemptive and living-donor transplants without generating net savings or affecting acute care utilization [23]. 

While CMS national evaluations have assessed aggregate model-level effects, evidence on health-system-level implementation remains limited [23]. As part of an overarching population health approach to managing expenditures and improving outcomes for patients with CKD, our health system implemented an array of care management interventions – called the Kidney Care Medical Home (KCMH) program – for patients with CKD in 2022 in response to CMS’ KCF Option. We hypothesized that the care management components of the program would be associated with reductions in emergency department (ED) and hospitalization utilization and improvements in process-of-care outcomes such as transplant referrals, behavioral health engagement, and home dialysis utilization. To this end we used advanced difference-in-difference (DiD) estimator methods to measure the time-varying impacts of the KCMH program on acute care utilization, use of home dialysis, and processes of care specific to the program compared with a contemporaneous control group from 2021 to 2023.

## Methods

The study setting was a large urban academic health system comprising over 800,000 unique patients, 5 hospitals, and 280 clinics. Our retrospective cohort study used the Callaway and Sant’Anna DiD estimator [26] to evaluate the impact of the KCMH program on utilization and outcomes over multiple timeframes from 1/1/2021 through 12/31/2023. These analyses compared the KCF enrolled cohort identified by CMS enrollment files to a similarly constructed [27] but unenrolled control cohort enrolled in Primary Care First (PCF), a Medicare FFS cohort for whom we had universal claims data. Specifically, a patient must have a diagnosis of CKD stage 4–5 or ESRD, have had at least two nephrology visits within the period of 15 to 3 months prior to the alignment date, and have had recent kidney function labs (e.g., creatinine, eGFR). Additionally, they must not meet any of the exclusion criteria: death, age under 18, in hospice, or have had a kidney transplant within the period of 13 to 1 month prior to the alignment date. These criteria mimic the KCF alignment logic set by CMS. Additionally, CMS’s program overlap policy prohibited concurrent alignment to PCF and KCF, making the cohorts mutually exclusive [27]. This work received Institutional Review Board (IRB) approval (IRB#23-001731), and, because this was an observational study of a health system led quality improvement initiative, the UCLA IRB issued a waiver of informed consent. Throughout this manuscript, KCF refers to the CMS payment model that defined the exposure, whereas the KCMH program refers to the local, health-system-designed care delivery intervention implemented in response to KCF participation.

### KCMH program

Exposure to the intervention was defined as enrollment in the KCF Option, as this prompted the KCMH program components outlined in Table 1. The control cohort was constructed from patients aligned with a PCF enrolled practice in which participating practices receive prospective population-based payments and flat primary care visit fees with performance-based adjustments tied to quality and utilization measures [28]. Control patients received usual primary and specialty care bolstered by system-wide population health efforts afforded to all primary care patients.

**Table 1:** Components of the kidney care medical home program

Component	Description	Target Population	Delivery Mode
Chronic Care Management	Care coordination with increased nursing touchpoints and regular telephone outreach aimed at reducing unnecessary ED visits and hospitalizations. The care management team consisted of RN care coordinators working with nephrologists, licensed clinical social workers, pharmacists, specialty care coordinators, and primary care providers Enhanced management for complex patients with comorbid congestive heart failure, includes weekly and quarterly check-ins, and educational resources, and remote monitoring of vital signs (e.g., body weight).	All KCF-enrolled patients; enhanced management for those with comorbid CHF and access to mobile devices	Telephone outreach by RN care coordinators; EHR-integrated remote monitoring
Depression and Patient Activation Screening (CMS-required)	Routine screening with the Patient Health Questionnaire-9 (PHQ-9) for depression and the Patient Activation Measure (PAM) for engagement and self-management readiness. Positive PHQ-9 screens prompt behavioral health referrals. Low PAM scores prompt care coordination outreach and rescreening within four months.	All KCF-enrolled patients	In-person or telemedicine by clinic staff during routine visits
Kidney Education Program (KEP) and Shared Decision-Making	Nurse-led education on kidney treatment options (e.g., home hemodialysis, transplant, conservative management, etc.) to prepare patients for CKD progression. The nurse would additionally promote shared decision-making between the patient and their provider.	All KCF-enrolled patients	In-person or telemedicine by a specially trained kidney nurse educator

### Data sources

Data were collected from Medicare Part A and Part B claims, Medicare KCF and PCF enrollment files containing beneficiary demographic and enrollment status, and internal data from the electronic medical record (EMR). The health system’s participation in both the KCF and PCF Innovation Models provided universal Part A and B claims for all patients enrolled in either program including utilization outside of the health system (i.e., leakage). Descriptive measures of intervention uptake, included to characterize program reach, were obtained from the EMR and from Phreesia, a CMS-selected vendor for PAM analysis.

### Study cohort

The treatment (KCF) population was constructed using the monthly attribution report provided by CMS between 1/1/2021 and 12/31/2023 identified per the program alignment and eligibility criteria [27]. The control population was constructed from the PCF cohort using similar eligibility criteria. Specifically, a patient must have a diagnosis of CKD stage 4–5 or ESRD, have had at least two nephrology visits within the period of 15 to 3 months prior to the alignment date, and have had recent kidney function labs (e.g., creatinine, eGFR). Additionally, they must not meet any of the exclusion criteria: death, age under 18 years, in hospice, or have had a kidney transplant within the period of 13 to 1 month prior to the alignment date.

### Outcome measures

The primary outcomes included all-cause ED utilization that did not result in hospitalization (treat and release) as well as all-cause hospitalization. Secondary outcomes included referrals to behavioral health resources, referrals to transplant nephrology, and the use of home dialysis. We also measured rates of ED or hospital utilization related to hypo- and/or hypervolemia because these are problems common among patients with CKD that are potentially sensitive to the high-intensity ambulatory care coordination and clinical monitoring provided by the program [29]. 

### Statistical analysis

We estimated the impact of the KCMH model on outcomes using a heterogeneous DiD design [26]. Given that patient enrollment in the intervention is staggered over time and treatment effects may vary over time, we used the Callaway and Sant’Anna estimator. This estimator is robust to biases of two-way fixed effects models, such as heterogeneity in treatment effects, and allows for flexibility in parallel trends conditional on covariates and how treatment effects are aggregated. We constructed ordinary least squares DiD models to quantify the average treatment effect on the treated (ATET). To calculate ATETs, we compared outcomes among the intervention group versus the control group pre- vs. post-intervention period in each quarter, assuming that outcome trends in the exposure group would parallel the control group without exposure to the intervention. Statistical testing for parallel pre-intervention trends was not used because these tests are not reliable for definitively ruling out significant violations of the parallel trends assumption [30, 31]. All analyses were completed in Stata 18 using built-in packages.

## Results

During the study period, we identified 926 KCF (treatment) patients and 551 control patients.

Patient demographic characteristics and prevalence of comorbidities were generally similar between treatment and control groups (Table 2). The mean age was similar between control (78.3 ± 14.7 years) and treatment groups (77.9 ± 11.6 years, *p* = 0.55). Sex distribution was also comparable, with females constituting approximately 46.2% of the control and 47.3% of the treatment groups (*p* = 0.491). Social vulnerability index (SVI) rankings were similar across both groups (control: 37.8 ± 26.8; treatment: 38.5 ± 27.0; *p* = 0.547). Comorbidity prevalence differed somewhat, with the control group exhibiting higher rates of dementia (7.0% vs. 2.3%) and depression (11.5% vs. 6.9%). Approximately 26% of treatment patients lacked an attributed in-network primary care provider (PCP), whereas all control patients had attributed PCPs. Complete mixed-effects Poisson regression output of demographics appear in Additional File 1 as Table S1.

**Table 2:** Demographic and clinical characteristics of treatment and control groups

Characteristics	Control	Treatment
Population	551	926
Age	78.25 (14.7)	77.89 (11.59)
Sex (%)		
Male	53.67	52.7
Female	46.17	47.3
Race (%)		
White	47.92	48.81
Black/African-American	11.5	7.45
Hispanic	15.34	12.1
Asian	9.74	13.28
Middle Eastern/North African	1.12	2.05
Native Hawaiian or Pacific Islander	0.48	0.43
American Indian or Alaska Native	0.32	0.54
Multiple	0.8	1.62
Unknown	12.78	13.71
SVI	37.80 (26.83)	38.51 (27.02)
Dual Eligibles (%)	29.87	29.1
Comorbidities		
Dementia	7.03	2.27
Depression	11.5	6.91
Uncomplicated Diabetes	25.4	23.76
Complicated Diabetes	34.19	34.88
Heart Failure	18.69	14.96
Uncomplicated Hypertension	56.23	50.97
Complicated Hypertension	13.58	15.01
Obesity	4.95	2.92
Has a health system PCP	98.72	74.3

Baseline characteristics of KCF-enrolled and control patients. Values presented as mean(standard deviation) for continuous variables and percentages for categorical variables

In the intervention group, baseline PAM completion was 70% in 2022 and 65% in 2023. Patients with PAM levels 1–3 (not fully activated, scores between 0 and 72.4) were engaged and rescreened demonstrating a mean 19.63 and 19.38 point increase in 2022 and 2023, respectively. Baseline PHQ-9 was completed for 55% and 53% of patients in 2022 and 2023, respectively.

Throughout the study period, intervention group patients visited the ED 1,006 times (9.25/100 patients/quarter) and were hospitalized 1,835 times (16.87/100 patients/quarter); the control group experienced 703 ED visits (10.86/100 patients/quarter) and 1,325 hospitalizations (20.45/100 patients/quarter).

**Fig. 1:**
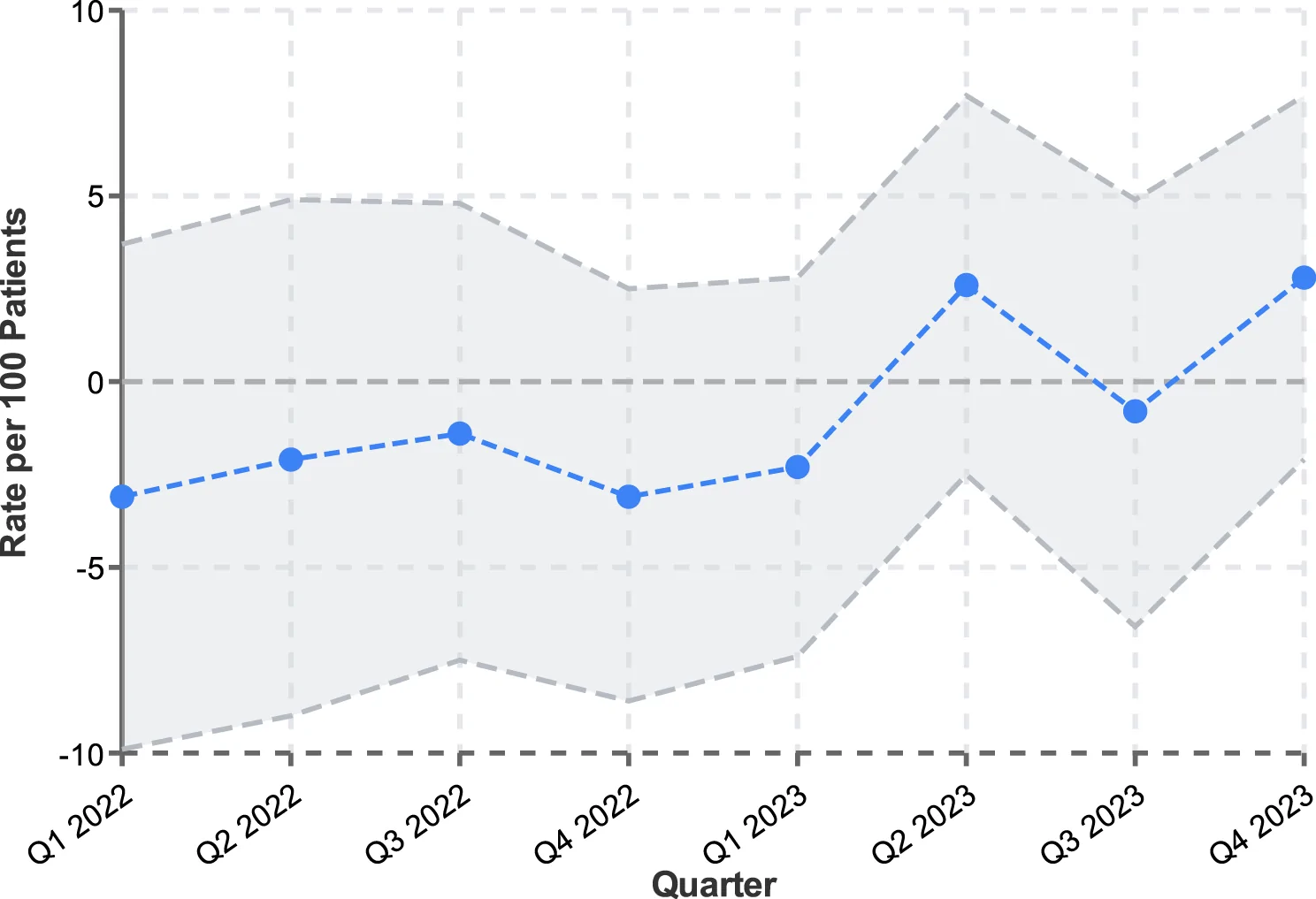
Average treatment effect on the treated for all-cause ED visits by Quarter, 2022–2023. Estimates derived using a difference-in-differences estimator comparing KCF enrolled patients to control patients. Models adjusted for age, sex, SVI, dual eligibility, and comorbidities. Error bars represent 95% confidence intervals. Dashed line at zero represents no treatment effect

**Fig. 2:**
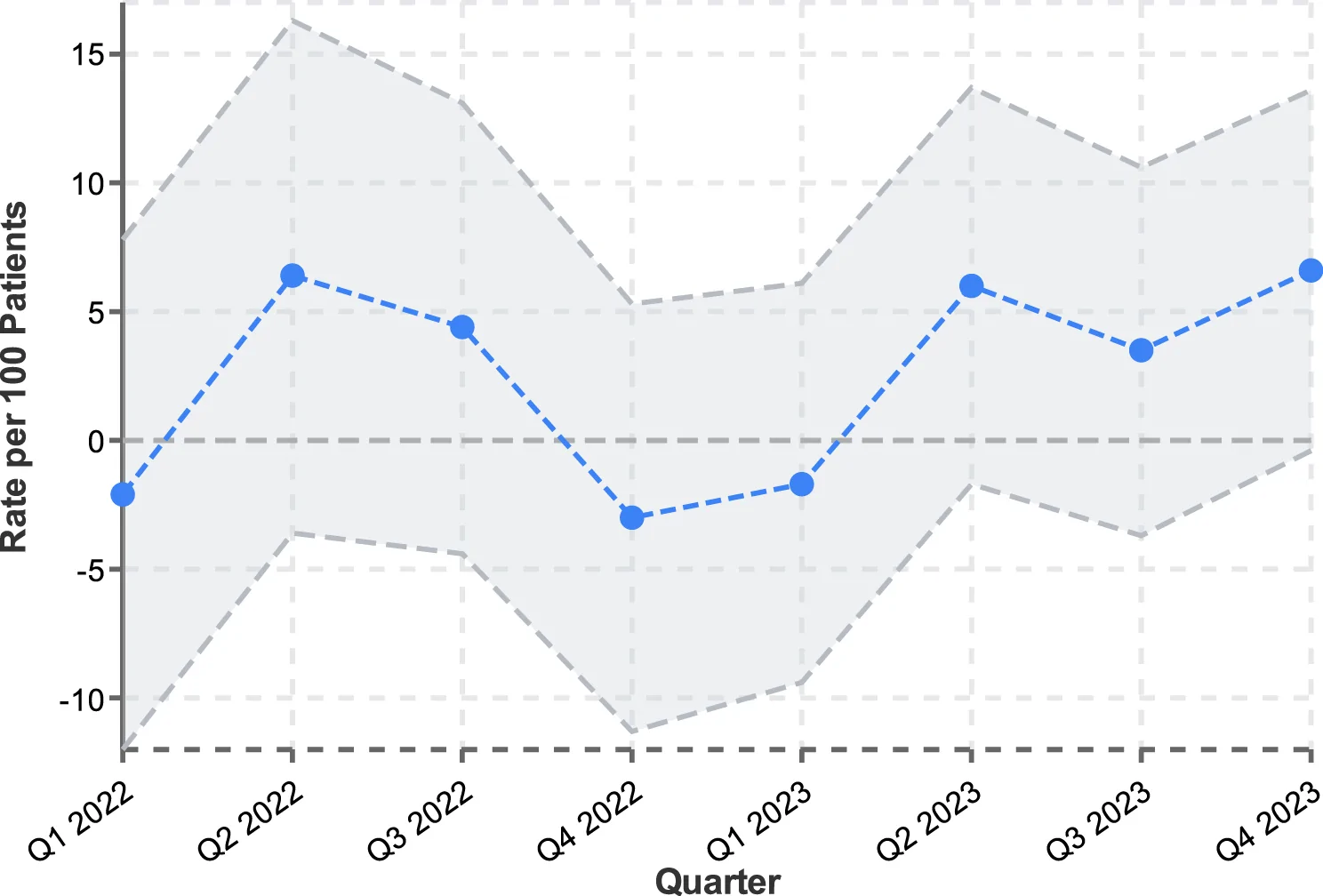
Average treatment effect on the treated for all-cause hospitalizations by Quarter, 2022–2023. Estimates derived using a difference-in-differences estimator comparing KCF enrolled patients to control patients. Models adjusted for age, sex, SVI, dual eligibility, and comorbidities. Error bars represent 95% confidence intervals. Dashed line at zero represents no treatment effect

In adjusted models controlling for age, sex, SVI, dual eligibility, and comorbidities, enrollment in KCF was not associated with statistically significant differences in the change in either all-cause ED visits or hospitalizations. Specifically, there was no significant difference in the change in quarterly ED visits, with ATET ranging from − 3.09 to + 2.80 visits per 100 patients (95% confidence interval (CI) [-9.90, + 7.66]) (Fig. 1), nor in inpatient hospitalizations, with quarterly ATET ranging from − 3.00 to + 6.60 visits per 100 patients (95% CI [-12.02, + 16.32]) (Fig. 2).

Secondary outcomes showed varied trends: home dialysis rates significantly increased in the later part of 2023, specifically Q3 (ATET = + 3.05; 95% CI, + 0.46 to + 5.65; *p* = 0.021) and Q4 (ATET = + 4.96; 95% CI, + 2.18 to + 7.74; *p* < 0.001) (see Additional File 1: Fig. S1). Transplant nephrology referrals significantly increased in 2022 Q3 (ATET = + 1.52; 95% CI, + 0.16 to + 2.88; *p* = 0.029) and Q4 (ATET = + 1.69; 95% CI, + 0.48 to + 2.90; *p* = 0.006), but no significant changes in 2023 (see Additional File 1: Fig. S2). Behavioral health assessment referrals significantly increased only in Q2 2022 (ATET = + 3.95; 95% CI, + 0.24 to + 7.67; *p* = 0.037), with other quarters showing no significant differences (see Additional File 1: Fig. S3).

Renal or volume overload-specific ED and inpatient visit analyses did not demonstrate any statistically significant differences across all evaluated quarters. ED visits related to renal conditions or overload ranged from ATET = -0.41 (95% CI, -2.02 to + 1.20; *p* = 0.614) to + 1.43 (95% CI, -0.77 to + 3.63; *p* = 0.204) (see Additional File 1: Fig. S4). Similarly, hospitalizations for renal or overload conditions ranged from ATET = -0.65 (95% CI, -3.89 to + 2.58; *p* = 0.691) to + 2.72 (95% CI, -0.85 to + 6.29; *p* = 0.135), with no significant quarterly differences (see Additional File 1: Fig. S5). Full ATET coefficient tables available in Additional File 1 as Tables S2-S8.

### Discussion

This population health implementation of a KCMH program in response to CMS’s KCF Option was not associated with a reduction in all-cause ED visits or hospitalizations. Despite robust care coordination efforts, the adjusted DiD estimates for utilization remained small and not statistically significant. However, the program was associated with several important improvements in secondary outcomes. Increases were seen in referrals to transplant nephrology, referrals to behavioral health resources, and home dialysis utilization; this pattern appeared to accelerate once the program was operationally mature. The concentration of transplant referral gains in 2022 may reflect the program addressing a backlog of referral-eligible patients at launch; with limited cohort turnover, incident referral need in 2023 may have been smaller, attenuating the incremental effect. The high rates of PAM and PHQ-9 completion, along with the large increase in PAM scores for lower scoring patients suggest strong patient engagement across multiple aspects of the program. These results suggest that while short-term acute care utilization proved difficult to change, the program was successful in engaging patients in other key components of their care that could lead to longer-term improvements in health, decrease in utilization, and decrease in total cost of care.

These results are broadly consistent with existing literature and analyses of CKD-focused value-based care. Notably, our inability to substantially reduce all-cause ED visits and hospitalizations is consistent with the national evaluation of the KCC model which also found no significant decrease in acute care hospitalization or ED visit rates among model participants [23]. However, these results contrast with earlier innovation models focused on ESRD, such as the CEC Model which reported a modest (~ 5%) reduction in hospitalizations and lower readmission rates (reduced by 1.8% points) [17]. One possible explanation for this discrepancy is that the CEC model targeted ESRD patients on dialysis with very high event base rates, whereas KCF encompasses a broader range of CKD where acute events are generally less frequent. The literature shows that the difficulties in reducing acute care utilization through care coordination are not unique to CKD and ESRD. Disease management programs for chronic disease have often failed to demonstrate significant short-term reductions in ED visits and hospitalizations, particularly when layered on to fee-for-service systems [32, 33]. 

This study also presents novel insights into the efficacy of secondary interventions not otherwise measured. We demonstrated significant increases in referrals to transplant nephrology and behavioral health under the program – outcomes that reflect the care coordination efforts and program-specific outreach. These process improvements have been less explored in earlier studies and underscore the value of comprehensive care models: by systematically screening for depression and educating patients on treatment options, KCF appears to have meaningfully improved aspects of care that are essential for patient-centered outcomes but often overlooked in traditional models.

Evaluating these data from a policy lens, our results highlight that even when financial incentives are aligned, achieving large changes in utilization remains challenging. Despite our population health approach and patient-centered focus, our institution remains predominantly fee-for-service in its reimbursement structure. In essence, KCF was layered onto a volume-driven, fee-for-service environment, unintentionally creating tension between the value-based objectives and existing financial structures. It may be that broader financial levers and stronger incentives are required to drive systemic change. While the health system allocated substantial resources to the program, aggressive outpatient management strategies come with high opportunity costs. This aligns with national observations that value-based kidney care models require significant upfront investments in staffing and infrastructure without immediate savings to compensate [3, 34]. Implementation science literature predict sustained adaptation periods before complex multicomponent interventions achieve their intended outcomes [35, 36]. Additionally, the finding that 25% of KCF patients were not attributed to an internal PCP suggests room to improve care coordination. This reflects a structural feature of KCF attribution that did not require system-level PCP attribution. Without PCP involvement, curbing care fragmentation and reducing associated utilization will remain elusive [4, 6–9]. In addition, system-level interventions aimed at primary care patients – some potentially in response to the PCF program underpinning the control group – may have been applied unevenly due to this discrepancy in primary care attribution. Future policy efforts might increase impact by better integrating primary care in their scope.

Despite the lack of impact on acute care utilization, the improvements observed in secondary outcomes are encouraging and suggest that some of the more directly actionable components of the model may carry implications for cost and outcomes over the longer term. By late 2023, KCF-enrolled patients were significantly more likely to initiate home dialysis, and throughout the intervention we saw higher rates of transplant referrals and depression screenings/referrals in the treatment group. These are positive developments that are associated with better outcomes at a lower cost. For instance, patients managed with peritoneal dialysis report higher quality of life in multiple domains compared to those on in-center hemodialysis [10]. Likewise, kidney transplantation confers a clear long-term survival benefit and cost advantage [2, 12]. The increases seen in home dialysis utilization and transplant evaluations in our program could translate into improved clinical outcomes and lower expenditures in the future, even if these did not manifest as reductions in ED or hospital events during the study period. We also consider the uptick in referrals to behavioral health to be meaningful given the high prevalence of depression among CKD patients and association with poorer quality of life, higher mortality, and higher costs [11]. 

Health systems and providers face a learning curve in new care paradigms, and short evaluation windows may not capture the full trajectory of improvement. As CMS continues to experiment with alternative payment models for CKD and ESRD, evidence from this pragmatic population health intervention can inform future initiatives. These findings underscore that positive effects may take years to realize and should prompt CMS to adopt longer-term impact analyses for innovation models that incentivize upfront health investments.

### Limitations

Our study does have limitations to consider. First, as a non-randomized, retrospective observational analysis, we are unable to definitively establish causality. We used an alternative DiD estimator robust to biases of two-way fixed effects models with a contemporaneous control cohort to account for secular trends and baseline differences, but unmeasured confounding variables remain possible. However, the consistency of our findings across multiple outcomes and time periods suggests that unmeasured confounding would need to be substantial and systematic to fully explain our results.

Second, while the control group was constructed from patients enrolled in PCF, and were not selected for the full suite of KCMH interventions, the nature of PCF does financially incentivize the health system to improve clinical quality and patient experience measures [37]. These incentives may have prompted concurrent interventions for primary care patients that cannot be entirely disentangled from our outcomes and could plausibly bias estimated treatment effects to the null, diluting the apparent effect of the treatment. The staggered design of our alternative DiD estimator is specifically designed to address this challenge, as PCF participants were only exposed to limited interventions during specific quarters. Notably, we did not identify a single quarter where the KCF cohort showed improved utilization relative to controls, suggesting that any PCF-related improvements did not substantially bias our findings.

Third, this was a single-center study at a large academic medical system, which may limit generalizability. These results may not apply to other systems, particularly fully-capitated systems where practice patterns and financial incentives differ.

Fourth, operational nuances in program implementation that are not fully captured by this quantitative analysis could affect outcomes. Like all novel care programs, the KCMH program took time to become fully operational at full capacity, and certain measures like referrals do not guarantee receipt of mental health services or transplants. However, our DiD estimator method is particularly well-suited to address this limitation, as it provides quarterly effect estimates that can capture the dynamic rollout of the intervention.

Fifth, the cohorts differed at baseline on PCP attribution due to each program’s alignment methodology: nephrology-based KCF alignment versus primary-care-based PCF attribution. We additionally noted some differences in the prevalence of dementia and depression; our models rely on a conditional parallel-trends assumption incorporating these measured covariates, but residual confounding from unmeasured time-varying differences in disease severity cannot be excluded. Because CMS reclaimed the model data following early termination of these Innovation Models, we were unable to further characterize any differences in the population, such as CKD stage distribution, eGFR, dialysis status, or incident versus prevalent dialysis, or outcomes, such as cardiovascular events or improvements in depression.

Finally, our study period may be insufficient to capture the full, long-term effects of the program. Some benefits of enhanced CKD care (such as slowing disease progression or preventing complications, and downstream effects on utilization) might only manifest over a longer period.

## Conclusions

In summary, the implementation of a KCF-aligned Kidney Care Medical Home program in a large academic health system was not associated with short-term reductions in ED visits or hospitalizations among Medicare beneficiaries with CKD stages 4–5 and ESRD. However, it was associated with increased engagement in important secondary care domains. These findings suggest that, while comprehensive CKD care models may face challenges in quickly curbing acute care utilization, they can achieve meaningful improvements in patient-centered domains that may accrue improvements in health outcomes over time. Our results provide evidence to inform future payment reforms targeting CKD: introducing generally aligned financial incentives and thoughtful care coordination may not be sufficient to drastically cut utilization in the short term, particularly in predominantly fee-for-service environments and where many patients did not have primary care involvement. Nevertheless, these models do encourage critical steps towards higher-value care. Health systems and policy makers can leverage these results to inform future value-based CKD/ESRD programs – expanding on successful elements such as incentivizing transplants, encouraging engagement with behavioral health, and strengthening patient education and engagement.

## Supplementary Information

Below is the link to the electronic supplementary material.

Supplementary Material 1: Additional file 1: Supplementary tables and figures. File format: .docx. Table S1: Mixed-effects Poisson regression output for demographic covariates. Table S2: ATET estimates for all-cause ED visits by quarter, 2022–2023. Table S3: ATET estimates for all-cause hospitalizations by quarter, 2022–2023. Table S4: ATET estimates for home dialysis sessions by quarter, 2022–2023. Table S5: ATET estimates for transplant nephrology referrals by quarter, 2022–2023. Table S6: ATET estimates for behavioral health referrals by quarter, 2022–2023. Table S7: ATET estimates for renal or volume overload-related ED visits by quarter, 2022–2023. Table S8: ATET estimates for renal or volume overload-related hospitalizations by quarter, 2022–2023. Fig. S1: ATET for home dialysis sessions by quarter, 2022–2023. Fig. S2: ATET for transplant nephrology referrals by quarter, 2022–2023. Fig. S3: ATET for behavioral health referrals by quarter, 2022–2023. Fig. S4: ATET for renal or volume overload-related ED visits by quarter, 2022–2023. Fig. S5: ATET for renal or volume overload-related hospitalizations by quarter, 2022–2023.

## Data Availability

The datasets analyzed for this current study are not publicly available due to the inclusion of protected health information and restrictions and data retention policies under the Medicare data use agreement. Data are however available from the authors upon reasonable request and with appropriate approvals and assistance of Medicare.

## Abbreviations

ACO: Accountable care organization
ATET: Average treatment effect on the treated
CEC: Comprehensive ESRD Care
CI: Confidence interval
CKD: Chronic kidney disease
CMS: Centers for Medicare and Medicaid Services
DiD: Difference-in-differences
ED: Emergency department
EMR: Electronic medical record
ESRD: End-stage renal disease
ETC: ESRD Treatment Choices
FFS: Fee-for-service
IRB: Institutional review board
KCC: Kidney Care Choices
KCF: Kidney Care First
KCMH: Kidney Care Medical Home
KEP: Kidney Education Program
PAM: Patient Activation Measure
PCF: Primary Care First
PCP: Primary care provider
PHQ-9: Patient Health Questionnaire-9
SVI: Social Vulnerability Index
