# Supplementary material for: Impact of a health system’s population health implementation of Medicare’s “Kidney Care First” program: a retrospective cohort study
Source: BMC Health Serv Res. 2026 Aug 17;26:1592. doi: 10.1186/s12913-026-15323-2 (PMC13760921; doi:10.1186/s12913-026-15323-2)
Supplement: Supplementary file 1 [file 12913_2026_15323_MOESM1_ESM.docx]

```
SUPPLEMENTARY MATERIALS

Table S1: Mixed-effects Poisson regression output for demographic covariates.

DEMOGRAPHICS REGRESSION OUTPUT
Mixed-effects Poisson regression  # Observations = 1,476
------------------------------------------------------------
             | Coeff.   SE      z      P>|z|   [95% CI]
-------------+----------------------------------------------
      female |   .168   .074    2.27   0.023    .023    .312
         age |  -.002   .003   -0.61   0.541   -.008    .004
dualeligible |   .322   .085    3.78   0.000    .155    .488
             |
ethnoracecat |
      Asian  |  -.431   .509   -0.85   0.396   -1.43    .565
   Black/AA  |   .173   .506    0.34   0.733   -.820    1.17
Hisp/Latino  |  -.003   .502   -0.01   0.996   -.987    .982
      ME/NA  |  -.111   .563   -0.20   0.843   -1.22    .993
   Multiple  |   .060   .581    0.10   0.918   -1.08    1.20
      NHOPI  |   .093   .691    0.13   0.893   -1.26    1.45
    Unknown  |  -.242   .506   -0.48   0.632   -1.23    .749
      White  |  -.059   .499   -0.12   0.905   -1.04    .919
             |
    dementia |   .037   .134    0.28   0.783   -.225    .299
  depression |   .440   .100    4.41   0.000    .244    .636
     diab_cx |   .155   .089    1.73   0.083   -.020    .330
   diab_uncx |  -.004   .091   -0.05   0.963   -.182    .174
heartfailure |   .248   .076    3.26   0.001    .099    .397
      htn_cx |   .002   .082    0.03   0.979   -.159    .163
    htn_uncx |  -.095   .073   -1.30   0.192   -.238    .048
     obesity |  -.076   .145   -0.53   0.598   -.361    .208
       _cons |  -2.90   .538   -5.38   0.000   -3.95   -1.84
------------------------------------------------------------
```

```
Table S2: ATET estimates for all-cause ED visits by quarter, 2022–2023.

ATET over time                        Number of obs = 17,367
    (Std. err. adjusted for 1,476 clusters in patientid)
------------------------------------------------------------
Time    |   ATET  Robust SE       z  P>|z|          [95% CI]
--------+---------------------------------------------------
Q1 2022 | -0.031      0.035  -0.890  0.374  -0.099     0.037
Q2 2022 | -0.021      0.036  -0.580  0.561  -0.090     0.049
Q3 2022 | -0.014      0.031  -0.430  0.664  -0.075     0.048
Q4 2022 | -0.031      0.028  -1.070  0.283  -0.086     0.025
Q1 2023 | -0.023      0.026  -0.880  0.381  -0.074     0.028
Q2 2023 |  0.026      0.026   0.990  0.320  -0.025     0.077
Q3 2023 | -0.008      0.029  -0.280  0.779  -0.066     0.049
Q4 2023 |  0.028      0.025   1.130  0.258  -0.021     0.077
------------------------------------------------------------
```

```
Table S3: ATET estimates for all-cause hospitalizations by quarter, 2022–2023.

ATET over time                        Number of obs = 17,367
    (Std. err. adjusted for 1,476 clusters in patientid)
------------------------------------------------------------
Time    |   ATET  Robust SE       z  P>|z|          [95% CI]
--------+---------------------------------------------------
Q1 2022 | -0.021      0.051  -0.420  0.677  -0.120     0.078
Q2 2022 |  0.064      0.051   1.260  0.209  -0.036     0.163
Q3 2022 |  0.044      0.045   0.980  0.329  -0.044     0.131
Q4 2022 | -0.030      0.042  -0.710  0.480  -0.113     0.053
Q1 2023 | -0.017      0.039  -0.420  0.672  -0.094     0.061
Q2 2023 |  0.060      0.039   1.530  0.126  -0.017     0.137
Q3 2023 |  0.035      0.037   0.940  0.346  -0.037     0.106
Q4 2023 |  0.066      0.036   1.860  0.063  -0.004     0.136
------------------------------------------------------------
```

```
Table S4: ATET estimates for home dialysis sessions by quarter, 2022–2023.

ATET over time                        Number of obs = 17,367
    (Std. err. adjusted for 1,476 clusters in patientid)
------------------------------------------------------------
Time    |   ATET  Robust SE       z  P>|z|          [95% CI]
--------+---------------------------------------------------
Q1 2022 | -0.014      0.009  -1.500  0.133  -0.032     0.004
Q2 2022 | -0.003      0.017  -0.180  0.858  -0.036     0.030
Q3 2022 | -0.006      0.021  -0.280  0.780  -0.048     0.036
Q4 2022 | -0.017      0.018  -0.970  0.332  -0.052     0.018
Q1 2023 | -0.004      0.016  -0.230  0.815  -0.034     0.027
Q2 2023 |  0.020      0.016   1.230  0.219  -0.012     0.051
Q3 2023 |  0.031      0.013   2.310  0.021   0.005     0.056
Q4 2023 |  0.050      0.014   3.490  0.000   0.022     0.077
------------------------------------------------------------
```

```
Table S5: ATET estimates for transplant nephrology referrals by quarter, 2022–2023.

ATET over time                        Number of obs = 17,367
    (Std. err. adjusted for 1,476 clusters in patientid)
------------------------------------------------------------
Time    |   ATET  Robust SE       z  P>|z|          [95% CI]
--------+---------------------------------------------------
Q1 2022 |  0.010      0.006   1.640  0.102  -0.002     0.023
Q2 2022 |  0.006      0.005   1.030  0.301  -0.005     0.016
Q3 2022 |  0.015      0.007   2.180  0.029   0.002     0.029
Q4 2022 |  0.017      0.006   2.740  0.006   0.005     0.029
Q1 2023 | -0.004      0.007  -0.520  0.602  -0.018     0.010
Q2 2023 |  0.001      0.005   0.200  0.843  -0.009     0.011
Q3 2023 |  0.006      0.005   1.250  0.212  -0.003     0.015
Q4 2023 | -0.002      0.005  -0.330  0.743  -0.011     0.008
------------------------------------------------------------
```

```
Table S6: ATET estimates for behavioral health referrals by quarter, 2022–2023.

ATET over time                      Number of obs = 17,367
   (Std. err. adjusted for 1,476 clusters in patientid)
----------------------------------------------------------
Time    |  ATET  Robust SE      z  P>|z|          [95% CI]
--------+-------------------------------------------------
Q1 2022 | 0.001      0.010  0.100  0.924  -0.018     0.020
Q2 2022 | 0.040      0.019  2.090  0.037   0.002     0.077
Q3 2022 | 0.005      0.010  0.560  0.579  -0.014     0.025
Q4 2022 | 0.005      0.005  1.120  0.262  -0.004     0.014
Q1 2023 | 0.003      0.007  0.410  0.679  -0.010     0.016
Q2 2023 | 0.004      0.008  0.540  0.591  -0.012     0.021
Q3 2023 | 0.008      0.005  1.400  0.160  -0.003     0.018
Q4 2023 | 0.008      0.004  1.760  0.079  -0.001     0.016
----------------------------------------------------------
```

```
Table S7: ATET estimates for renal or volume overload-related ED visits by quarter, 2022–2023.

ATET over time                      Number of obs = 17,367
    (Std. err. adjusted for 1,476 clusters in patientid)
------------------------------------------------------------
Time    |   ATET  Robust SE       z  P>|z|          [95% CI]
--------+---------------------------------------------------
Q1 2022 |  1.152      0.919   1.255  0.210  -0.648     2.953
Q2 2022 |  0.964      1.009   0.956  0.339  -1.013     2.942
Q3 2022 |  1.427      1.123   1.271  0.204  -0.774     3.629
Q4 2022 |  0.786      0.859   0.915  0.360  -0.897     2.469
Q1 2023 |  1.013      0.618   1.640  0.101  -0.198     2.224
Q2 2023 | -0.414      0.821  -0.504  0.614  -2.023     1.195
Q3 2023 |  0.975      0.731   1.332  0.183  -0.459     2.408
Q4 2023 |  0.500      0.721   0.693  0.488  -0.914     1.913
```

------------------------------------------------------------

```
Table S8: ATET estimates for renal or volume overload-related hospitalizations by quarter, 2022–2023.

ATET over time                      Number of obs = 17,367
    (Std. err. adjusted for 1,476 clusters in patientid)
------------------------------------------------------------
Time    |   ATET  Robust SE       z  P>|z|          [95% CI]
--------+---------------------------------------------------
Q1 2022 | -0.539      1.855  -0.291  0.771  -4.174     3.096
Q2 2022 |  1.800      1.964   0.916  0.360  -2.050     5.649
Q3 2022 |  2.720      1.820   1.495  0.135  -0.847     6.288
Q4 2022 | -0.655      1.649  -0.397  0.691  -3.887     2.577
Q1 2023 | -0.122      1.549  -0.079  0.937  -3.159     2.915
Q2 2023 |  0.768      1.441   0.533  0.594  -2.057     3.593
Q3 2023 |  0.668      1.425   0.469  0.639  -2.126     3.462
Q4 2023 |  1.310      1.360   0.963  0.335  -1.355     3.976
```

Fig. S1: ATET for home dialysis sessions by quarter, 2022–2023.

Fig. S2: ATET for transplant nephrology referrals by quarter, 2022–2023.

Fig. S3: ATET for behavioral health referrals by quarter, 2022–2023.

Fig. S4: ATET for renal or volume overload-related ED visits by quarter, 2022–2023.

Fig. S5: ATET for renal or volume overload-related hospitalizations by quarter, 2022–2023.
